# Fatty acid metabolism and lipid channeling in macrophages: mechanisms of inflammation, resolution, and lipotoxicity

**DOI:** 10.3389/fimmu.2026.1839069

**Published:** 2026-06-12

**Authors:** Temitayo T. Bamgbose, Oluwakemi O. Igiehon, Javier Nion-Fieira, Cora R. Palmer, Andrii Andriichuk, Matthew D. Woolard

**Affiliations:** 1 Department of Microbiology and Immunology, Louisiana State University Health Sciences Center, Shreveport, LA, United States; 2 Center for Applied Immunology and Pathological Processes (CAIPP), Louisiana State University Health Sciences Center, Shreveport, LA, United States

**Keywords:** fatty acid metabolism, inflammation resolution, lipid channeling, lipotoxicity, macrophages

## Abstract

Macrophages integrate metabolic signals with immune activation, and their ability to handle fatty acids is central to preventing lipotoxicity and to sustaining effector functions. In cardiometabolic settings such as obesity, metabolic dysfunction-associated steatotic liver disease, and atherosclerosis, chronic exposure to excess free fatty acids and toxic lipid species, such as ceramides, disrupts organelle integrity, impairs efferocytosis, and skews macrophages toward pro-inflammatory phenotypes. This review examines how macrophages channel fatty acids into β-oxidation, glycerolipid synthesis and storage, sphingolipid production, and polyunsaturated fatty acid-derived lipid mediator biosynthesis to shape the balance among metabolic adaptation, inflammatory activation, resolution, and lipid-induced dysfunction. We also highlight lipin-1 as a regulatory node at a key branchpoint in macrophage lipid metabolism. By linking glycerolipid synthesis, lipid storage, mitochondrial metabolism, and inflammatory signaling, lipin-1 illustrates how lipid routing can influence macrophage function in cardiometabolic disease.

## 1 Introduction

**Figure 1:**
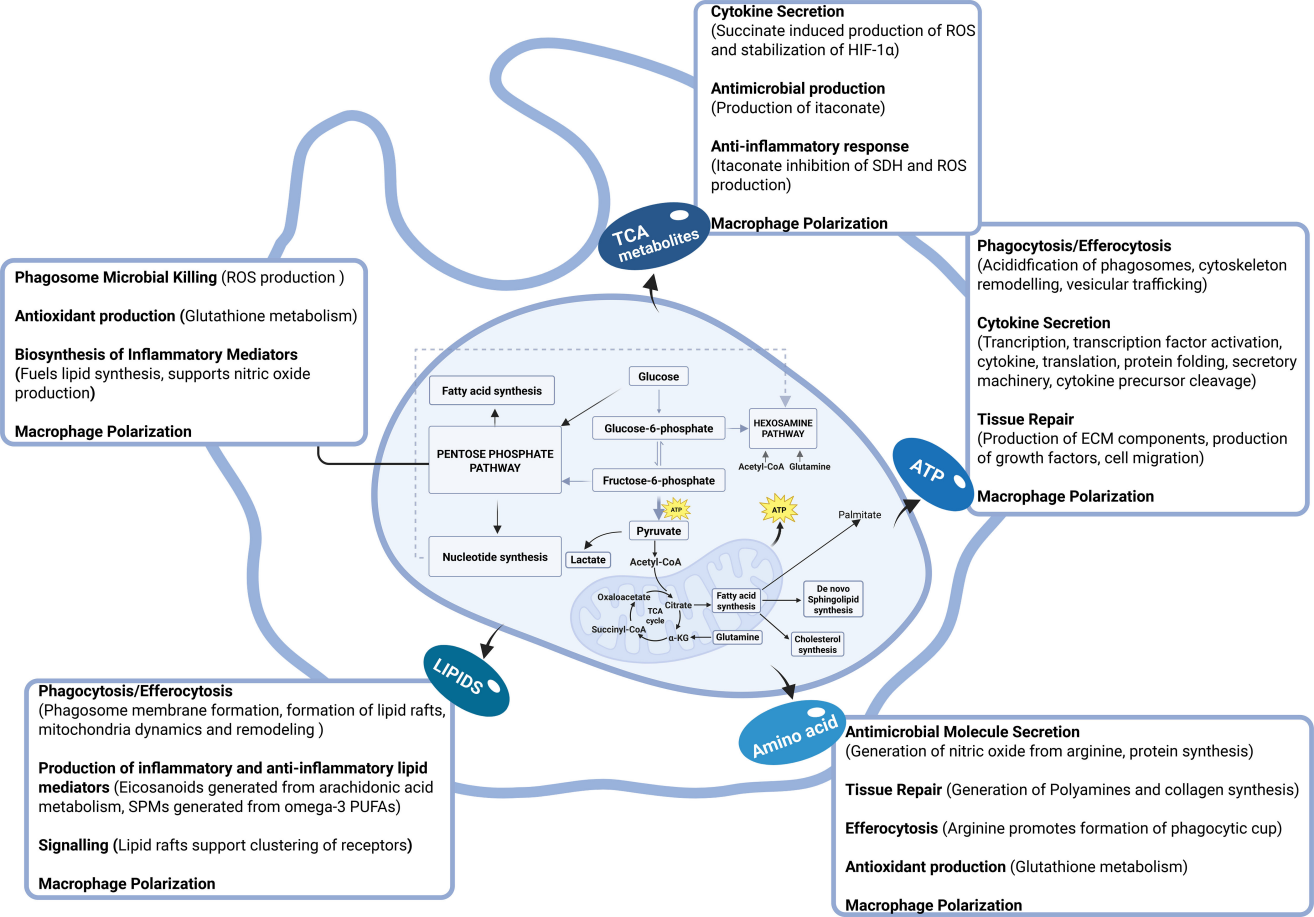
Core metabolic pathways that support macrophage effector function. Macrophage effector functions are supported by interconnected metabolic pathways, including glucose, lipid, and amino acid metabolism, which generate ATP and biosynthetic intermediates required for inflammatory signaling, phagocytosis, efferocytosis, and tissue repair. In this review, we focus specifically on how lipid metabolism and fatty acid channeling shape these responses.

Macrophages are central regulators of tissue homeostasis, host defense, and repair. They respond to cytokines, dying cells, extracellular matrix cues, and nutrient availability, adopting functional states that shape pathogen clearance, inflammatory signaling, efferocytosis, and tissue remodeling. Immunometabolism has provided an important framework for understanding these responses. Pro-inflammatory macrophages commonly increase glycolysis and reprogram the TCA cycle and mitochondrial electron transport chain to support rapid cytokine production, reactive oxygen species (ROS) generation, and antimicrobial activity, whereas pro-resolving macrophages more often engage in mitochondrial respiration, fatty acid oxidation, and biosynthetic programs linked to efferocytosis and tissue repair (**Figure 1**). The contributions of glycolysis, the TCA cycle, and oxidative phosphorylation have been extensively reviewed and will not be discussed in detail here. Throughout this review, we refer to pro-inflammatory and pro-resolving macrophages rather than M1 and M2 macrophages because these terms better capture macrophage functional programs and avoid oversimplifying macrophage activation states.

Lipid metabolism has emerged as a major determinant of macrophage function. Free fatty acids (FFAs) serve not only as energy sources and membrane precursors, but also as regulators of membrane composition, organelle integrity, redox balance, inflammatory signaling, and macrophage effector function. However, because unesterified FFAs can be cytotoxic at high concentrations, macrophages must tightly channel FFAs into defined metabolic fates (1, 2). Thus, the impact of FFAs on macrophages is not determined by lipid abundance alone. Instead, macrophage responses depend on how FFAs are taken up, activated, stored, oxidized, or converted into bioactive lipid species. These intracellular routing decisions help determine whether lipid exposure supports metabolic adaptation and effector function or promotes inflammatory activation and lipotoxic dysfunction. In this review, we examine how *de novo* fatty acid synthesis and fatty acid channeling regulates macrophage biology, with emphasis on pathways that control β-oxidation, glycerolipid metabolism, lipid mediator production, and ceramide generation. We also highlight lipin-1 as a regulatory node at a key branchpoint in macrophage lipid metabolism, illustrating how lipid handling can influence inflammatory and pro-resolving macrophage responses in cardiometabolic disease.

## 2 Routing fatty acids to shape macrophage function

Macrophage responses to lipid-rich environments are determined not only by the availability of fatty acids but by how these substrates are routed within the cell. This section discusses our current mechanistic understanding of fatty acid flux and lipid channeling in macrophages, focusing on how fatty acids are taken up, trafficked, activated, and directed into distinct metabolic fates that influence cellular function. In this review, we use the term lipid channeling to describe the movement of FFAs through different pathways within the cell (3, 4). This can occur either passively, when fatty acids follow pathways shaped by enzyme abundance and metabolic demand, or actively, when fatty acid-binding proteins, membrane contact sites, and enzyme complexes help direct lipids to specific locations. After activation to fatty acyl-CoA intermediates, fatty acids can enter mitochondrial β-oxidation, glycerolipid synthesis and storage, sphingolipid and ceramide biosynthesis, and the generation of polyunsaturated fatty acid (PUFA)-derived lipid mediator pathways, including prostanoid and resolvin production. These pathways can have distinct and sometimes opposing effects on macrophage inflammatory activation, efferocytosis, tissue repair, and susceptibility to lipotoxic stress.

### 2.1 Fatty acid uptake, synthesis, trafficking, and activation

**Figure 2:**
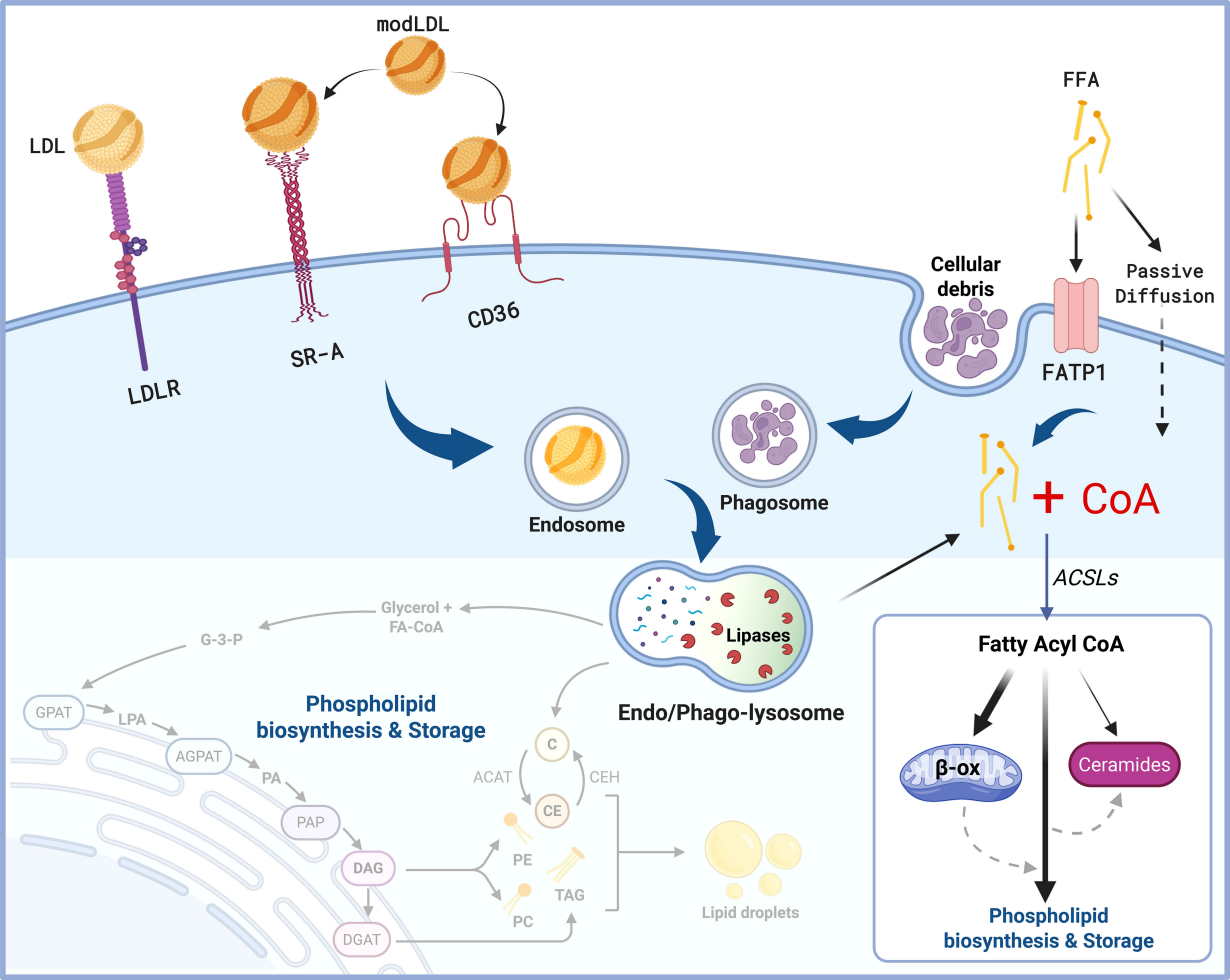
Fatty acid uptake and intracellular channeling in macrophages. Macrophages acquire lipids through LDLR-mediated uptake of LDL, scavenger receptor-mediated uptake of modified lipoproteins, FATP1-dependent import of FFAs, passive diffusion, and lysosomal liberation of fatty acids from internalized lipoproteins or phagocytic cargo. Following uptake or intracellular liberation, FFAs are converted by acyl-CoA synthetases into fatty acyl-CoAs, which are then directed into mitochondrial β-oxidation, glycerolipid synthesis with storage in lipid droplets, or sphingolipid synthesis to generate ceramides.

Macrophages have a high capacity for lipid uptake and processing because they express multiple receptors and transport proteins that mediate the acquisition of extracellular lipids. These include low-density lipoprotein receptor (LDLR), which internalizes low-density lipoproteins (LDL) particles, scavenger receptors such as CD36 and scavenger receptor type A (SRAs), which bind a broad range of modified lipids and lipoproteins, fatty acid transport protein 1 (FATP1), which mediates uptake of long-chain fatty acids, and triggering receptor expressed on myeloid cells 2 (TREM2), a lipid-sensing receptor that helps coordinate macrophage lipid handling in lipid-rich environments (5–8). Through these uptake pathways, intracellular fatty acids arise from receptor-mediated uptake of lipoproteins, direct import of FFAs, lysosomal liberation of fatty acids from internalized lipoproteins or phagocytic/efferocytic cargo, or passive diffusion (**Figure 2**).

In addition to acquiring fatty acids from extracellular sources, macrophages can generate fatty acids through *de novo* synthesis. When energy is abundant, citrate is exported from mitochondria through the mitochondrial citrate carrier and cleaved by ATP citrate lyase to generate acetyl-CoA, which is then converted by acetyl-CoA carboxylase into malonyl-CoA, the first committed intermediate in fatty acid biosynthesis (9, 10). Fatty acid synthase (FASN) then uses malonyl-CoA to generate palmitate, which can then be activated (11). Thus, macrophages generate intracellular fatty acid pools through both extracellular uptake and endogenous synthesis.

Once inside the cell, FFAs are bound by fatty acid-binding proteins (FABPs), which facilitate their distribution to distinct intracellular compartments and metabolic pathways (12, 13). Regardless of origin, whether from exogenous uptake or endogenous liberation, FFAs are typically converted to fatty acyl-CoAs by acyl-CoA synthetases, which generate the activated intermediates used in β-oxidation and complex lipid synthesis (3). In some cases, uptake pathways may also influence downstream routing; for example, CD36 has been proposed to contribute to mitochondrial fatty acid delivery through its interaction with carnitine palmitoyltransferase I (CPT1), thereby enhancing β-oxidation (8, 14).

These early steps in fatty acid handling have important consequences for macrophage function. *De novo* fatty acid synthesis provides lipids needed for membrane remodeling, inflammatory signaling, and effector function, but its role is not limited to pro-inflammatory activation (15, 16). Inhibition of acetyl-CoA carboxylase (ACC), the first committed step in *de novo* fatty acid synthesis, blunts LPS-induced phospholipid accumulation, ROS production, bactericidal activity, and pro-inflammatory cytokine secretion, while FASN inhibition reduces pro-inflammatory macrophage recruitment, promotes anti-inflammatory responses, and limits inflammation-driven disease severity (17). Mechanistically, FASN-generated palmitate supports TLR4 signaling by promoting Akt palmitoylation, phosphorylation, and membrane recruitment, thereby facilitating MAPK activation; these responses are reduced when FASN is inhibited (18, 19). However, lipid synthesis also supports pro-resolving macrophage functions beyond cell viability (20). TLR4 activation induces a biphasic lipid synthesis program, with early production of inflammatory fatty acids followed by later SREBP-dependent production of anti-inflammatory unsaturated fatty acids that suppress NF-κB activity, while TLR4-driven mTORC1 activation stimulates SREBP-1a-dependent lipid synthesis required for phagocytosis (21, 22).

After uptake, intracellular trafficking, and activation, fatty acyl-CoA is channeled into several major metabolic fates. First, fatty acyl-CoA can be conjugated to carnitine and transported into mitochondria for β-oxidation, thereby supporting ATP production via the electron transport chain. Second, fatty acyl-CoA can be incorporated into glycerolipid synthesis, with excess neutral lipids subsequently packaged into lipid droplets that serve as storage organelles and hubs of lipid metabolism. Third, fatty acyl-CoAs can contribute to the synthesis of PUFA-derived lipid mediators, including prostanoids and specialized pro-resolving mediators (SPMs) such as resolvins. Fourth, fatty acyl-CoAs can enter *de novo* sphingolipid biosynthesis, where they are coupled to amino acids such as serine to generate ceramides. Each of these pathways shapes macrophage function in distinct ways and will be discussed in the following sections.

### 2.2 β-oxidation

β-oxidation is the catabolism of fatty acyl-CoAs and occurs in both peroxisomes and mitochondria. In mammalian cells, peroxisomes primarily process very long-chain fatty acids, whereas mitochondria oxidize short-, medium-, and long-chain fatty acids (23). Mitochondrial import of long-chain fatty acids requires CPT1 and CPT2, after which fatty acids undergo sequential reactions in the mitochondrial matrix to generate acetyl-CoA, citric acid cycle intermediates, and electron carriers that support electron transport and ATP production (24). This pathway is especially important in macrophages, which must manage high lipid burdens generated by uptake of bacteria, parasites, cellular debris, and dead or dying cells. By catabolizing fatty acids from exogenous and endogenous sources, β-oxidation supports energy production, limits intracellular lipid accumulation, and helps protect macrophages from lipotoxicity (4, 20).

Early models linked alternatively activated or pro-resolving macrophage programs to β-oxidation-coupled oxidative phosphorylation, but this view has become increasingly nuanced. Macrophages can engage glycolysis, glutamine metabolism, or fatty acid oxidation depending on the stimulus, tissue environment, substrate availability, and effector function being performed (25, 26). Although the requirement for β-oxidation in pro-resolving macrophage polarization remains debated, current evidence suggests that IL-4-driven macrophage polarization can occur independently of β-oxidation (27–29). This metabolic flexibility is further supported by the ability of pro-resolving macrophages to use glycolysis-derived pyruvate, glutamine, or fatty acid-derived acetyl-CoA to sustain TCA cycle activity and oxidative phosphorylation (26, 30–33). In contexts where fatty acids are available and β-oxidation is engaged, this pathway can generate acetyl-CoA to support TCA cycle activity, electron carrier production, and oxidative phosphorylation (34–38). Thus, β-oxidation should not be viewed as a definitive feature of pro-resolving macrophage identity. Instead, in contexts where fatty acids are available and β-oxidation is engaged, this pathway can provide acetyl-CoA and support mitochondrial respiration needed for selected effector functions. Functionally, β-oxidation and oxidative phosphorylation have been shown to enhance uptake of dying cells and promote IL-10 production during efferocytosis through pathways involving the electron transport chain, NAD+, and SIRT1-dependent signaling (36, 39). Collectively, these findings indicate that although β-oxidation may not be required for canonical IL-4-driven macrophage polarization, it provides important metabolic support for pro-resolving macrophage effector functions, particularly efferocytosis, IL-10 production, and tissue repair.

### 2.3 Lipid storage

Once FFAs are converted to fatty acyl-CoAs, they can be incorporated into complex lipids, including cholesterol esters, diacylglycerols, phospholipids, and triacylglycerols (TAGs). Lipid droplets are dynamic intracellular organelles that store neutral lipids, TAGs and cholesteryl esters, within a hydrophobic core surrounded by a phospholipid monolayer and associated proteins (40). In macrophages, however, lipid droplets are not simply passive lipid reservoirs. They also support immune function by storing newly synthesized neutral lipids and modulating membrane remodeling, inflammatory mediator production, and phagocytic activity (41, 42). Consistent with their protective role, inhibition of DGAT-dependent lipid droplet formation increases lipotoxic stress and can promote macrophage cell death (43). Thus, lipid droplet formation represents a key fate of activated fatty acids, linking lipid storage, protection from lipotoxicity, and macrophage effector function.

Lipid droplet biogenesis is closely linked to neutral lipid synthesis at the endoplasmic reticulum. As intracellular lipid content increases, TAGs and cholesteryl esters accumulate between the two leaflets of the ER membrane, allowing nascent lipid droplets to bud from the ER (44–46). TAGs are generated through the glycerolipid/Kennedy pathway, in which fatty acyl-CoAs are sequentially esterified onto a glycerol backbone to form phosphatidic acid (PA), and then diacylglycerol (DAG). The terminal step is catalyzed by diacylglycerol acyltransferases, DGAT1 and DGAT2, which convert DAG and fatty acyl-CoA into TAG. Functionally, DGAT-dependent TAG synthesis is crucial for TLR-driven inflammatory activation in macrophages, as its inhibition restrains the formation of lipid droplets, the production of downstream inflammatory mediators such as PGE_2_, IL-1, and IL-6, and even phagocytosis (15). In contrast, DGAT-dependent TAG synthesis in murine macrophages attenuated FFA-induced lipotoxicity and inflammatory activation in diet-induced obesity (1).

To couple lipid metabolism with immune effector functions, macrophages rely on lipid droplet turnover, which includes the tightly regulated, overlapping pathways of cytosolic lipolysis and lipophagy. Lipolysis is the neutral enzymatic hydrolysis of TAGs and cholesterol esters to generate FFAs and glycerol through a stepwise and highly controlled enzymatic cascade. First, adipose triglyceride lipase (ATGL) hydrolyzes TAG into DAGs and FFAs at the cytosolic surface of lipid droplets. Subsequently, hormone-sensitive lipase (HSL) hydrolyzes DAGs into FFAs and MAGs. Finally, MAGs are hydrolyzed by monoacylglycerol lipase (MAGL) into glycerol and additional FFAs (47, 48). In parallel with rapid cytosolic lipid droplet lipolysis, macrophages employ lipophagy, a specialized and selective form of autophagy. Unlike stepwise enzymatic lipolysis at the lipid droplet surface, lipophagy typically mediates lipid droplet turnover by sequestering entire lipid droplets or their fragments into LC3-positive autophagosomes. These autophagosomes subsequently fuse with lysosomes, where lysosomal acid lipase (LAL) hydrolyzes neutral lipids, enabling a more controlled and sustained release of FFAs that is coordinated with autophagic flux and lysosomal signaling (49, 50).

Although cytosolic lipolysis has been proposed to precede lipophagy, with neutral lipases acting on large lipid droplets to reduce their size and facilitate autophagic engulfment, this sequential model remains debatable. Lipophagy can be activated independently of prior lipolysis, especially during nutrient deprivation (51, 52). Regardless of the route of lipid droplet degradation, lipid droplet turnover can release FFAs that are redirected toward β-oxidation and PUFAs that serve as substrates for prostanoid and specialized pro-resolving mediator synthesis (52–54). Furthermore, these lipolytic and lipophagic products engage lipid-sensing pathways and nuclear receptors such as PPARs, which govern the transcriptional regulation of cytokine production and macrophage polarization (55). Together, these pathways highlight lipid droplet lipolysis and lipophagy as central metabolic nodes linking lipid droplet dynamics to both pro-inflammatory and pro-resolving macrophage responses (6). However, the consequences of lipid droplet turnover are context-dependent, as lipolysis and lipophagy can either induce or restrain macrophage oxidative stress and cell death (56, 57).

### 2.4 PUFA-derived lipid mediators

PUFAs are precursors for bioactive lipid mediators that regulate both the initiation and resolution of inflammation. Following release from cellular lipid pools, including membrane phospholipids and lipid droplets, PUFAs such as arachidonic acid (AA), eicosapentaenoic acid (EPA), and docosahexaenoic acid (DHA) can be enzymatically converted into distinct classes of lipid mediators (58). AA-derived eicosanoids, including prostaglandins and leukotrienes, generally promote inflammatory signaling, leukocyte recruitment, and cytokine production (59). In contrast, omega-3 PUFAs, including EPA and DHA, give rise to specialized pro-resolving mediators (SPMs) such as resolvins, protectins, and maresins, which actively counter-regulate inflammation and promote tissue repair (60–62). Thus, PUFA metabolism represents a major branch of fatty acid channeling in which related lipid substrates can be routed toward either inflammatory or pro-resolving mediator production.

The biological effects of PUFA-derived mediators depend on the enzymatic pathways engaged and the receptors activated. AA released from membrane phospholipids by phospholipase A2 can be converted by cyclooxygenase enzymes into prostaglandin intermediates, which are then processed by specific synthases to generate prostaglandins such as PGE_2_ (63). COX-1 is constitutively expressed, whereas COX-2 is inducible during inflammatory activation, and mPGES1 has been linked to inducible PGE_2_ production in macrophages (64). In parallel, lipoxygenase pathways convert AA into leukotrienes and also contribute to the generation of SPMs. EPA- and DHA-derived SPMs are generated through coordinated lipoxygenase-, COX-2-, and cytochrome P450-dependent pathways, often involving transcellular biosynthesis among immune and stromal cells (58). These pathways are important because they do not simply produce “inflammatory” versus “anti-inflammatory” lipids. Rather, they generate mediator families with distinct timing, receptor usage, and biological effects during inflammation and resolution. Pro-inflammatory eicosanoids can sustain macrophage activation through pathways such as NF-κB signaling, inflammasome activation, and cytokine production, whereas SPMs signal through G-protein-coupled receptors, including ALX/FPR2, ChemR23, and GPR32, to actively reprogram macrophage responses (65, 66).

These lipid mediator pathways have direct consequences for macrophage function. Pro-inflammatory eicosanoids promote cytokine production and inflammatory activation and can interfere with efficient efferocytosis by disrupting receptors and signaling mechanisms required for apoptotic cell clearance, including MerTK-dependent pathways. In contrast, SPMs suppress NF-κB signaling and pro-inflammatory cytokine production, promote cytoskeletal rearrangements required for engulfment, enhance MerTK-dependent efferocytosis, and support metabolic programs associated with resolution, including oxidative phosphorylation (60–62, 67). Through these coordinated effects, SPMs shift macrophages toward a pro-resolving phenotype, facilitating the clearance of apoptotic cells and the restoration of tissue homeostasis. Failure to maintain this balance results in persistent inflammation, defective clearance mechanisms, and progressive tissue damage. Thus, PUFA-derived lipid mediator biosynthesis illustrates how fatty acid channeling can generate divergent macrophage outcomes, with inflammatory eicosanoid production sustaining activation and SPM biosynthesis promoting resolution.

### 2.5 Sphingolipid and ceramide synthesis as a lipotoxic branchpoint

Ceramide biosynthesis is another pathway into which activated fatty acids are channeled. In *de novo* sphingolipid biosynthesis, palmitoyl-CoA is first coupled to serine, and subsequent ceramide synthase-catalyzed N-acylation of sphinganine also utilizes fatty acyl-CoA, making this pathway an additional sink for excess lipid substrates (68). Although sphingolipids are essential membrane components and bioactive signaling molecules, their synthesis must be tightly controlled because excessive accumulation, particularly of ceramides, is cytotoxic (69, 70). In fact, ceramides have been implicated as potent inducers of inflammation, especially in conditions of lipid overload and metabolic stress. In type 2 diabetes, LDL-derived plasma ceramides accumulate in macrophages and activate JNK and NF-κB inflammatory signaling, resulting in increased expression of pro-inflammatory mediators such as TNF-α, CCL2, IL-6, and reduced expression of the anti-inflammatory cytokine, IL-10 (71). Similarly, ceramide accumulation promotes NLRP3-dependent caspase-1 inflammasome activation in macrophages, thereby linking sphingolipid stress to obesity-associated inflammation and insulin resistance (72). Recent work demonstrated that IL-10 signaling limits the accumulation of saturated very-long-chain (VLC) ceramides to suppress the expression of inflammatory genes in macrophages (73).

Consistent with this, ceramides impair mitochondrial function and promote cellular dysfunction and apoptosis (68, 74, 75). Although relatively few studies have examined ceramide synthesis in macrophages, available evidence indicates that ceramides promote macrophage death and impair pro-resolving macrophage polarization and efferocytosis (76–79). Mechanistically, uptake of ceramides from aggregated LDL (agLDL) alters macrophage morphology and disrupts actin polymerization (77). Because actin polymerization is required for phagocytic cup formation, both exogenous and endogenous ceramides impair efferocytosis by disrupting membrane ruffle formation and inhibiting Rac1 recruitment to the plasma membrane (76, 79).

Collectively, these pathways illustrate that macrophage responses to fatty acids are determined not simply by lipid availability, but by how intracellular lipids are taken up, trafficked, activated, and routed among competing metabolic fates (**Table 1**). Channeling fatty acyl-CoAs into β-oxidation can support mitochondrial metabolism, ATP generation, and protection from lipid overload, whereas incorporation into glycerolipids and lipid droplets can provide membrane substrates, buffer excess fatty acids, and regulate inflammatory mediator production. In parallel, PUFA mobilization can generate eicosanoids and specialized pro-resolving mediators that shape inflammatory activation, efferocytosis, and resolution, while diversion into sphingolipid pathways can promote ceramide accumulation, mitochondrial dysfunction, impaired cytoskeletal remodeling, defective efferocytosis, and lipotoxic stress. Thus, fatty acid routing is not a passive consequence of lipid excess, but a regulated process that determines whether macrophages adapt to lipid-rich environments, amplify inflammatory signaling, or engage programs required for resolution and tissue repair.

**Table 1:** Summary of fatty acid channeling pathways in macrophages.

Lipid channel	Key molecular components	Primary outputs	Functional consequences in macrophages	Relevance to inflammation and disease
β-oxidation	CPT1, CPT2, acyl-CoA dehydrogenases, TCA cycle enzymes	Acetyl-CoA, NADH, FADH_2_, ATP	Supports efferocytosis, promotes IL-10 production, limits lipid accumulation	Supports resolution of inflammation; impaired β-oxidation contributes to lipid accumulation and defective clearance in atherosclerosis and metabolic disease
Glycerolipid synthesis and lipid droplet formation	ACSLs, GPAT, AGPAT, lipin-1 (PAP activity), DGAT	Phospholipids, DAG, TAG, lipid droplets	membrane remodeling, DAG-mediated inflammatory signaling, and lipid buffering	Promotes inflammatory activation when coupled to signaling pathways; protects against lipotoxicity but can contribute to foam cell formation in atherosclerosis
Lipid droplet mobilization	ATGL, HSL, lipophagy, autophagy machinery	fatty acids from stored TAGs	Source of FAs for oxidation or signaling; metabolic adaptation during activation and efferocytosis	Dysregulation can lead to lipid overload or insufficient energy supply, contributing to macrophage dysfunction in chronic inflammation
PUFA-derived lipid mediator biosynthesis	PLA2, COX, LOX, CYP450	Prostaglandins, leukotrienes, resolvins, protectins, maresins	Regulates inflammatory signaling, cytokine production, efferocytosis, and tissue repair	Determines balance between chronic inflammation and resolution; dysregulation contributes to persistent inflammation in obesity, MASLD/MASH, and atherosclerosis
Sphingolipid and ceramide synthesis	SPTLCs CerS Sphingomyelinases	Ceramides, complex sphingolipids	Promotes mitochondrial dysfunction, ER stress, apoptosis, and impaired efferocytosis; disrupts cytoskeletal dynamics and phagocytic capacity	Drives lipotoxicity, inflammation, and macrophage death; contributes to necrotic core formation in atherosclerosis and tissue injury in metabolic disease

This table highlights the major fatty acid channeling routes, their underlying molecular mechanisms, primary metabolic or lipid-derived outputs, effects on macrophage function, and contributions to inflammation and cardiometabolic disease.

## 3 From adaptation to lipotoxicity: when lipid handling fails

Building on the framework of lipid channeling described above, this section examines how dysregulated fatty acid routing influences macrophage function in the context of cardiometabolic disease. When lipid uptake and intracellular handling exceed the capacity for balanced channeling into oxidative, storage, and signaling pathways, macrophages accumulate bioactive lipid species that promote lipotoxic stress, inflammatory activation, and impaired resolution. We first consider the general cellular consequences of macrophage lipotoxicity and then examine how these processes manifest in specific disease contexts, including obesity, metabolic dysfunction-associated steatotic liver disease/metabolic dysfunction-associated steatohepatitis (MASLD/MASH), and atherosclerosis.

### 3.1 Cellular consequences of macrophage lipotoxicity

Excessive intracellular accumulation of FFAs, termed lipotoxicity, can promote pro-inflammatory responses, disrupt cellular homeostasis, induce organelle dysfunction, and ultimately induce cell death in macrophages (69, 70, 80). Intracellular fatty acid excess can arise from the release of fatty acids from lipid droplets (81), increased uptake driven by elevated extracellular FFA levels, or excessive lipid biosynthesis (70). When lipid burden exceeds the capacity for β-oxidation and safe storage, bioactive intermediates such as DAGs and ceramides accumulate and promote cellular dysfunction (70, 82–84). Excess ceramides and DAGs promote ER stress, reactive oxygen species production, and activation of pro-inflammatory signaling cascades (68–70, 83). Additionally, excessive fatty acid delivery to mitochondria can permeabilize mitochondrial membranes, uncouple respiration, and reduce membrane potential (69, 85–87). In total, excess FFAs can promote oxidative damage, lipid peroxidation, and pro-apoptotic signaling (44, 69, 70, 83). Together, these metabolic disturbances shift macrophages toward dysfunction, thereby amplifying inflammation, impairing clearance of cellular debris, and delaying resolution.

Beyond effects on viability, lipid overload and organelle stress have direct immunologic consequences in macrophages. Excess saturated fatty acids can induce lysosomal damage and inflammasome-linked IL-1β production, thereby linking lipid overload to inflammatory amplification in metabolic disease (88). In parallel, lipid droplet biogenesis and TAG synthesis serve as an adaptive buffering axis that is tightly integrated with macrophage activation, including regulation of IL-1β production, eicosanoid output, and phagocytic capacity (15). Because efficient inflammation resolution requires effective efferocytosis and production of pro-resolving lipid mediators, disruption of lipid catabolism can delay the transition from inflammatory to pro-resolving responses (15, 88–90). Accordingly, failure to efficiently transition from inflammatory to pro-resolving macrophage responses may contribute to the persistent, low-grade inflammation that characterizes many cardiometabolic disorders.

### 3.2 Obesity and adipose inflammation

**Figure 3:**
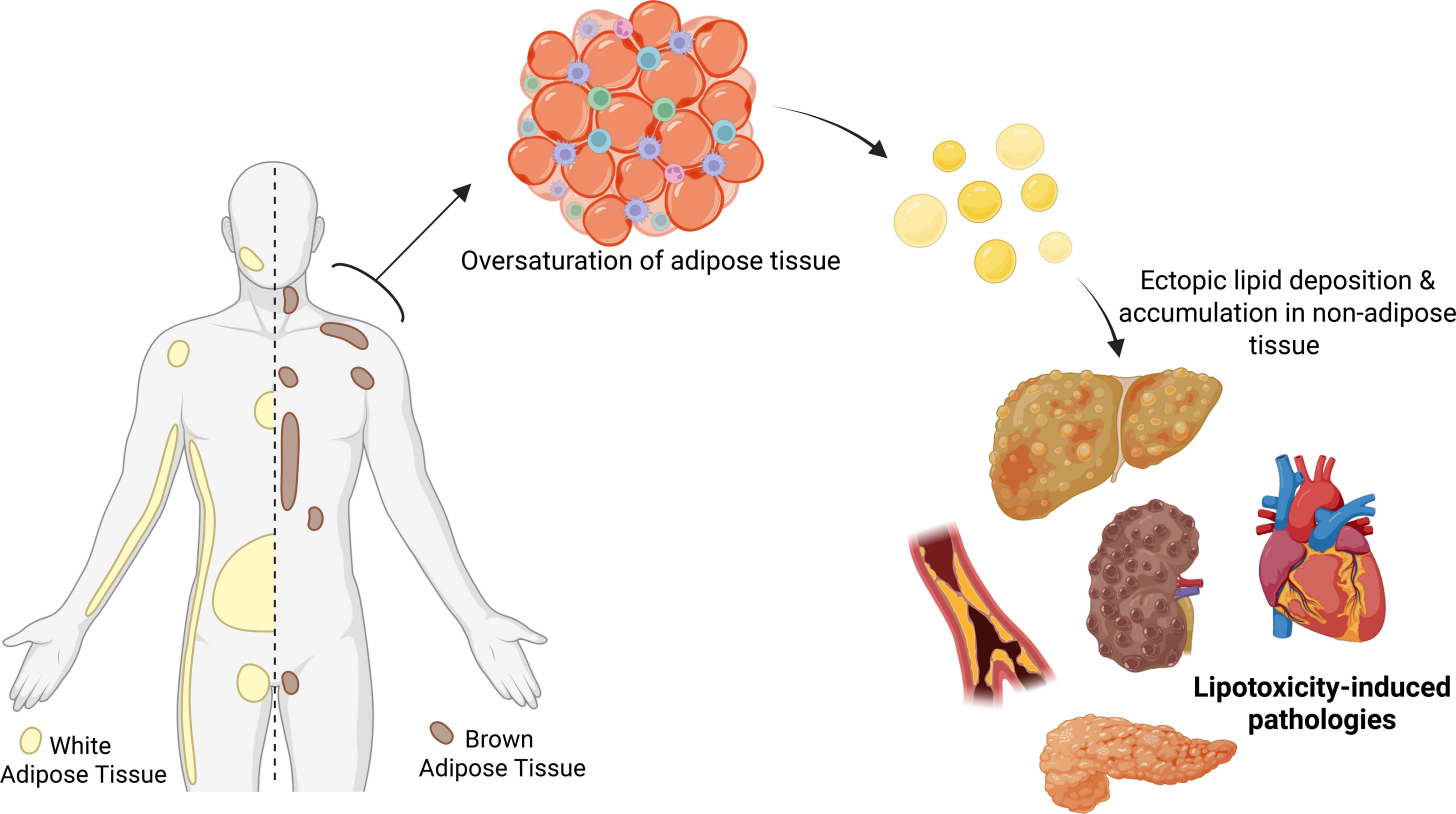
Finite adipose lipid-buffering capacity promotes ectopic lipid deposition and inflammation. White adipose tissue serves as the major site of lipid storage, but its buffering capacity is finite. When this capacity is exceeded during obesity, excess lipids spill into the circulation and accumulate in non-adipose tissues. This ectopic lipid deposition promotes tissue dysfunction, inflammation, and pathologies such as atherosclerosis, pancreatic steatosis, renal lipotoxicity, and steatotic liver disease.

In addition to the systemic effects of dyslipidemia and excess lipid burden on metabolism, circulation, and organ function, the accumulation of bioactive lipid species within macrophages promotes pro-inflammatory activation, sustaining chronic low-grade inflammation and contributing to cardiometabolic disease (**Figure 3**). This shift in macrophage activation can further disrupt tissue and whole-body metabolism and thereby contribute to insulin resistance and metabolic syndrome (91–97).

Obesity is a major driver of adipose macrophage lipid loading, lipotoxic stress, and chronic adipose inflammation because chronic nutrient excess is initially buffered by storage within adipose tissue, but adipose expandability is finite (98). When storage capacity is exceeded, excess fatty acids spill into the circulation, accumulate in non-adipose tissues, and promote lipotoxic pathology (98, 99). Adipose tissue expansion in obesity is accompanied by increased lipolysis and impaired lipid-buffering capacity, which promotes ectopic fatty acid spillover that can be partially buffered by adipose tissue macrophages (ATMs) (99, 100). ATMs help maintain adipose homeostasis by clearing dead adipocytes and scavenging excess lipids (101). However, sustained lipid spillover and adipocyte death impose metabolic stress on ATMs, promoting lipotoxicity and pro-inflammatory activation (101–103). ATMs are major contributors to adipose inflammation and insulin resistance, as depletion of phagocytic cells in high-fat diet-induced obesity reduces inflammation and improves insulin sensitivity (104). Inflammation within adipose tissue further sustains macrophage recruitment and ATM retention, creating a feed-forward cycle that is amplified by crosstalk between ATMs and T cells and by pro-inflammatory cytokine production from ATMs (105, 106). Together, these findings identify obesity as a state in which finite adipose storage capacity converts ATM lipid-buffering functions into a driver of chronic adipose inflammation and systemic metabolic dysfunction.

ATMs form crown-like structures around dying and necrotic adipocytes, where they engage in exophagy and take up large amounts of lipid and adipocyte debris (107). These ingested lipids are subsequently stored in lipid droplets, giving ATMs a foam cell-like appearance. Efficient lipid channeling is therefore particularly important in ATMs. Consistent with this, ATMs from lean and obese individuals differ in lipid metabolism (108). ATMs in obesity favor lipogenic programs that support the production of pro-inflammatory mediators, whereas ATMs in lean tissue more readily catabolize internalized fatty acids through β-oxidation (109, 110). Supporting a role for lipid synthesis in adipose inflammation, myeloid deletion of FAS in a high-fat diet-induced diabetes model improves insulin sensitivity by reducing inflammation and ATM recruitment to adipose tissue (111). Altered balance of PUFA-derived lipid mediators may also contribute to persistent low-grade inflammation in obese adipose tissue. Expanding adipose depots are enriched in inflammatory macrophages, and inadequate engagement of SPM-mediated resolution programs may sustain cytokine production, impair tissue repair, and contribute to metabolic dysfunction (112). Importantly, these ATM responses are heterogeneous and cannot be fully captured by a simple reparative-to-pro-inflammatory binary. Consistent with progressive lipid loading and metabolic stress, ATM populations in obesity become increasingly heterogeneous and include metabolically activated states that combine lipid-handling functions with inflammatory outputs rather than fitting neatly into classical polarization categories (110, 113). Notably, metabolically activated ATMs express lysosome-associated programs, promote inflammation, and participate in the clearance of dead adipocytes through lysosomal exocytosis, highlighting that ATMs responses to obesity reflect specialized adaptations to lipid-rich, damaged tissue environments rather than a simple shift between canonical activation states (114). Thus, in obesity, ATM function is shaped not only by lipid burden but also by how internalized fatty acids are channeled, stored, and metabolically processed within a heterogeneous macrophage population.

### 3.3 MASLD and MASH

MASLD is a prevalent liver disease in industrialized nations, and macrophages play central roles in its pathophysiology (115). In addition to hepatocytes, resident macrophages (Kupffer cells) and recruited monocyte-derived macrophages are major responders to excess lipid in the hepatic milieu. Fatty liver is associated with the accumulation of toxic lipid species such as DAGs and ceramides, which disrupt macrophage lipid homeostasis (116, 117). Excess lipid delivery promotes pro-inflammatory activation of Kupffer cells, and excessive uptake of cholesterol derived from oxidized LDLs (oxLDL) can convert them into fat-laden, foam cell-like macrophages that further drive hepatic inflammation and injury (118, 119). Consistent with this, reducing hepatic oxLDL content alleviates inflammation, whereas deletion of oxLDL receptors reduces hepatic injury and oxidative stress in Kupffer cells.

Macrophage scavenger receptor 1 (MSR1) is associated with increased accumulation of lipid-laden macrophages and correlates positively with the degree of steatosis and steatohepatitis in MASLD (120). In a MASLD model using global Msr1 knockout mice, MSR1 deficiency protected against diet-induced metabolic dysfunction, with fewer hepatic foamy macrophages, less hepatic inflammation, improved dyslipidemia and glucose tolerance, and altered hepatic lipid metabolism (120). Uptake of saturated fatty acids through macrophage-associated MSR1 also promotes inflammatory activation through JNK signaling (121). Accordingly, inhibition or blockade of MSR1 reduces macrophage lipid accumulation, limits the shift toward a pro-inflammatory phenotype, and decreases production of pro-inflammatory cytokines such as TNF-α (120, 121).

In addition to MSR1, triggering receptor expressed on myeloid cells 2 (TREM2) has emerged as a biomarker and potential therapeutic target in MASLD/MASH and related cardiometabolic diseases (122). Recent studies identified TREM2 as an important regulator of macrophage lipid handling, inflammatory responses, and disease progression in steatotic liver disease (123, 124). During MASH, bone marrow-derived TREM2-expressing macrophages are recruited to the liver, where they accumulate at sites of hepatocellular injury, inflammation, and fibrosis (122). Increased accumulation of these TREM2+ macrophages was associated with protection against MASH progression, whereas Trem2 deficiency in mice led to impaired lipid handling and extracellular matrix remodeling, resulting in exacerbated steatohepatitis, cell death, and fibrosis (122).

Macrophages, particularly Kupffer cells, are major producers of TNF in MASLD/MASH, and macrophage-derived TNF contributes to progression from steatosis to steatohepatitis and fibrotic liver injury (125, 126). When gut-derived LPS reaches the liver, it activates Toll-like receptor 4 (TLR4) on Kupffer cells, inducing a pro-inflammatory response. This response is further amplified when hepatic macrophages are exposed to both excess lipids and LPS, a condition that also promotes lipotoxic stress. Consistent with this, combined treatment of macrophages with LPS and palmitate synergistically increases ceramide production, and this TLR4-dependent response induces IL-1β and TNF expression (92, 127). IL-1β further promotes hepatic inflammation and steatosis, in part by suppressing PPARα signaling and thereby impairing fatty acid β-oxidation, increasing lipid accumulation, and amplifying pathways that drive liver injury and fibrosis (109). Dysregulated PUFA metabolism and reduced SPM production are associated with hepatic inflammation, lipid accumulation, and progression from steatosis to steatohepatitis (67, 128). SPMs have been shown to reduce inflammatory cytokine production, improve macrophage polarization, and enhance resolution pathways in the liver, thereby limiting disease progression (67, 129). Together, these findings suggest that in MASLD/MASH, maladaptive macrophage lipid routing toward lipid accumulation, inflammatory signaling, and impaired catabolic responses contribute to persistent hepatic inflammation and tissue injury.

### 3.4 Atherosclerosis

In addition to promoting pro-inflammatory macrophage activation, accumulation of toxic lipid derivatives can impair macrophage programs required for inflammation resolution, which are required to limit the progression of cardiometabolic disease (62, 94). Processes such as efferocytosis and anti-inflammatory cytokine secretion are essential for tissue repair and for limiting necrotic cell accumulation. In atherosclerosis, efficient clearance of dead cells and cellular debris by macrophages reduces necrotic core formation and promotes plaque stability, whereas impaired resolution sustains chronic inflammation (62, 130). This balance between pro-inflammatory eicosanoids and pro-resolving lipid mediators is particularly relevant in atherosclerosis, where chronic inflammation reflects not only persistent immune activation but also defective resolution. Impaired SPM biosynthesis and signaling contribute to sustained plaque macrophage activation, defective efferocytosis, and expansion of the necrotic core, whereas restoration of SPM pathways enhances apoptotic cell clearance and promotes a pro-resolving macrophage phenotype (61, 62, 106, 131).

Atherosclerosis underlies multiple cardiometabolic diseases, and macrophages are central to its pathophysiology. Following recruitment to sites of endothelial dysfunction, both monocyte-derived macrophages and resident intimal macrophages take up modified LDL and form foam cells that contribute to inflammatory processes within the plaque (132). Excessive lipid accumulation can also induce lipotoxic stress, leading to macrophage death and release of inflammatory mediators that promote necrotic core formation and plaque progression (133). Macrophages that do not become foamy can still adopt a highly inflammatory phenotype through expansion of cholesterol-rich lipid rafts, or “inflammarafts,” which prolong signaling by receptors such as TLR4 and other inflammatory receptor complexes (134, 135). Consistent with this, deletion of apolipoprotein A-I binding protein (AIBP) promotes accumulation of macrophages with increased lipid content and increased TLR4 inflammaraft expression, which is associated with larger lesions and necrotic cores. Further supporting a role for aberrant lipid biosynthesis in plaque progression, deletion of FASN in macrophages reduces foam cell formation and attenuates atherosclerosis severity (136).

We and others have shown that the downstream consequences of macrophage lipotoxicity contribute to atherosclerosis progression. Resveratrol, a natural polyphenol and PPAR agonist, reduces cholesterol and neutral lipid accumulation in oleate-treated macrophages and is associated with decreased intestinal fatty acid and monoglyceride accumulation in atherosclerotic mice. Mechanistically, resveratrol limits lipid loading in macrophages through PPAR-dependent induction of ABCA1- and ABCG1-mediated cholesterol efflux (137). Similarly, in foam-cell macrophages, lipophagy-derived free cholesterol can activate LXR/RXR-dependent transcription of ABCA1, thereby promoting cholesterol export and attenuating atherosclerosis (138; 139). Increased macrophage angiotensin-converting enzyme (ACE) expression promotes PPARα activity, increases ABCA1 and ABCG1 abundance, resulting in enhanced cholesterol efflux (137, 140). Thus, the ability to effectively move lipids within macrophages results in a protective response. In atherosclerotic settings, macrophage ACE expression has been linked to improved lipid handling and resolution-associated functions. Notably, constitutive macrophage ACE expression reduces atherosclerosis severity and promotes a CD36-expressing pro-resolving-like macrophage phenotype with increased β-oxidation, oxidative metabolic capacity, efferocytosis, and lipid uptake (141). Collectively, these observations support the idea that macrophage pathways favoring lipid efflux, oxidative metabolism, and pro-resolving functions are protective, whereas failed lipid handling promotes inflammatory signaling and plaque progression.

Atherosclerosis regression has gained increasing attention with the demonstration that intensive statin therapy and PCSK9 inhibitors can reduce human plaque burden over approximately 19 to 24 months (48, 142, 143). Under regression conditions, macrophage-derived cells emigrate from lesions, in contrast to the retention observed in progressive plaques (144, 145). Subsequent studies showed that HDL promotes regression in hAI/apoE-/- recipients and that macrophages remaining within atherosclerotic lesions display reduced inflammatory mediator expression and enrichment of a pro-resolving phenotype. This phenotypic shift is associated with increased secretion of anti-inflammatory mediators such as IL-10 and TGF-β, enhanced tissue remodeling and repair, and improved clearance of dead or dying cells through efferocytosis (146, 147). These effects were associated with improved macrophage efferocytotic capacity and a shift toward a more pro-resolving state in plaque macrophages. Whether these pro-resolving macrophages arise through phenotypic conversion of inflammatory macrophages or through replacement by newly recruited populations remains unresolved. Collectively, these findings suggest that macrophages capable of avoiding or overcoming lipotoxic dysfunction are important determinants of atherosclerosis regression.

## 4 Lipin-1 at the crossroads of macrophage lipid metabolism

The preceding sections highlight that macrophage responses to lipid excess are determined not only by fatty acid availability but by how these substrates are routed among competing metabolic and signaling pathways. Channeling fatty acids into β-oxidation, glycerolipid synthesis, lipid mediator production, or sphingolipid pathways can differentially support efferocytosis, inflammatory signaling, or lipotoxic stress. A central unresolved question is how macrophages coordinate these competing fates in a context-dependent manner to maintain function while avoiding dysfunction. Addressing this requires identifying regulatory nodes that integrate lipid flux with metabolic and immune signaling at key metabolic branchpoints. Among lipid metabolic regulators, lipin-1 is well positioned to serve as such a node. As a phosphatidic acid phosphatase that controls the conversion of phosphatidic acid to DAG, lipin-1 governs a central branchpoint in glycerolipid metabolism while also functioning as a nonenzymatic regulator of metabolic gene expression. Through these combined activities, lipin-1 links lipid synthesis, utilization, and inflammatory signaling within a shared regulatory framework. Emerging evidence further suggests roles in mitochondrial metabolism and organelle organization, processes central to the balance between adaptation and lipotoxicity. Thus, lipin-1 provides a biologically relevant example of how lipid channeling may be coordinated in macrophages and offers a framework for moving from descriptive models of fatty acid routing toward a more integrated understanding of macrophage function in inflammation and disease.

### 4.1 Structure and multifunctionality of lipin-1

**Figure 4:**
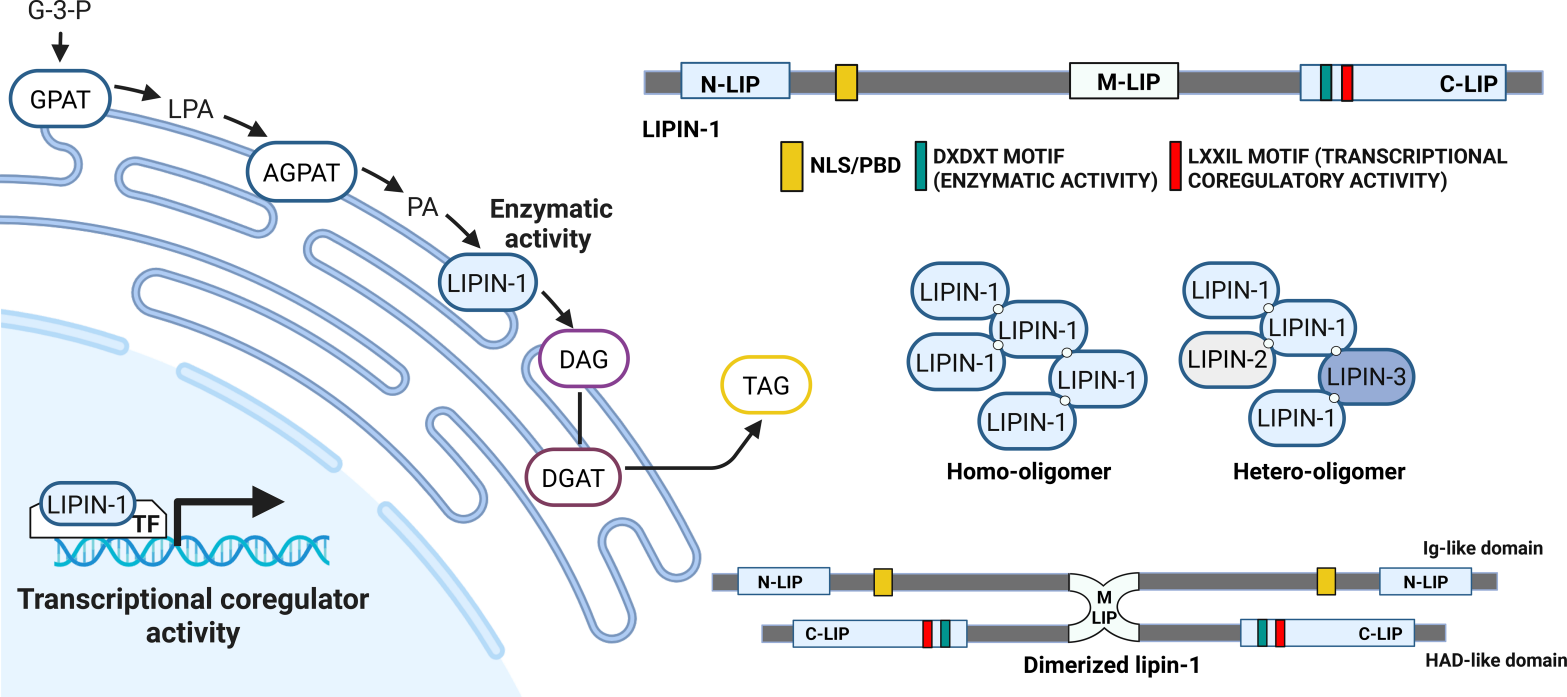
Structure and multifunctionality of lipin-1. **(A)** Lipin-1 functions as a Mg^2+^-dependent phosphatidic acid phosphatase that converts PA to DAG, thereby supporting TAG and phospholipid synthesis. Lipin-1 also functions as a transcriptional coregulator that promotes fatty acid catabolism through interactions with PPARα, PPARγ, and PGC-1α. **(B)** Lipin-1 contains conserved N-LIP and C-LIP regions that support catalytic and regulatory functions, as well as an M-LIP domain that contributes to membrane association and dimerization. **(C)** Lipin-1 forms homo-oligomers and hetero-oligomers with other lipin family members, including lipin-2 and lipin-3.

Lipin-1 is an evolutionarily conserved member of the lipin family that has two major functions: phosphatidic acid phosphatase activity, which converts phosphatidic acid to DAG during glycerolipid synthesis, and nonenzymatic transcriptional coregulatory activity that influences metabolic gene expression (**Figure 4**) (148–150). Structurally, lipin-1 contains conserved N-LIP and C-LIP regions that support catalytic and regulatory functions, as well as an M-LIP domain that contributes to membrane association, dimerization, and formation of lipin-1 homo- and hetero-oligomers with other lipin family members (151–153). In macrophages, this functional duality is especially important because lipin-1 can couple glycerolipid metabolism to inflammatory signaling while also supporting nonenzymatic programs linked to metabolic adaptation and pro-resolving function.

Among the best-defined regulatory features of lipin-1 is the linkage between phosphorylation, subcellular localization, and function. Insulin and mTORC1 signaling promote lipin-1 phosphorylation and cytoplasmic retention, thereby limiting its nuclear activity, whereas dephosphorylation favors redistribution and is associated with increased nonenzymatic regulatory functions (151, 154). Lipin-1 can also be redirected to the ER under lipid-rich conditions, indicating that localization helps determine whether lipin-1 primarily engages enzymatic or nonenzymatic functions (151, 155). Additional post-translational modifications that affect lipin-1 trafficking or stability have been reported, but their roles appear to be context-dependent and are less clearly defined (155, 156). Thus, the most established framework is that lipin-1 activity depends on both its dual molecular functions and its regulated intracellular localization.

### 4.2 Lipin-1 in macrophage function

Lipin-1 is a Mg2+-dependent phosphatidic acid phosphatase (PAP) within the glycerol 3-phosphate pathway that converts PA to DAG, thereby controlling a key branchpoint in glycerolipid synthesis (150). In macrophages, this enzymatic activity has been shown, particularly in TLR-activated and lipid-loaded macrophages, to support signaling pathways that amplify inflammatory responses under specific activation conditions (**Figure 4**). By increasing intracellular DAG, lipin-1 links lipid synthesis to pro-inflammatory signaling. During TLR4 activation, lipin-1-derived DAG contributes to MAPK and AP-1 activation and promotes expression of inflammatory mediators, consistent with a role for lipin-1 enzymatic activity in coupling glycerolipid synthesis to macrophage inflammatory responses (35, 157, 158). Lipin-1-derived DAG has also been linked to ER-localized TRPC3-dependent Ca2^+^ signaling during LPS stimulation (159–161). A central mechanistic theme emerging from these studies is that, under inflammatory or lipid-loading conditions, lipin-1 enzymatic activity generates signaling-competent DAG that can amplify downstream inflammatory pathways. Importantly, emerging evidence suggests that these pro-inflammatory outcomes may not reflect a direct intrinsic property of lipin-1 enzymatic activity, but rather the engagement of lipin-1 within broader signaling networks activated under inflammatory conditions. For example, perturbations of regulatory pathways upstream of lipin-1, including those involving SIRT1 and AMPK (162, 163), can enhance inflammatory signaling in macrophages, indicating that lipin-1-dependent effects on inflammation are highly context-dependent and integrated with other signaling modules.

Atherogenic lipid loading further reveals a role for lipin-1 enzymatic activity in foam cell biology, where it couples exposure to modified LDL to glycerolipid remodeling and sustained inflammatory signaling. In RAW264.7 macrophages treated with oxLDL or acetylated-LDL, lipin-1 contributes to DAG accumulation, foam-cell lipid loading, and production of pro-atherogenic mediators, including TNF-α, IL-6, and PGE_2_ (157). Mechanistically, in bone marrow-derived macrophages, lipin-1 enzymatic activity sustains a persistent DAG-responsive PKCα/βII-ERK1/2-cJun signaling cascade in response to modified LDL, indicating that lipin-1 converts enhanced glycerolipid synthesis into prolonged pro-inflammatory signaling rather than merely supporting passive lipid storage (158). *In vivo*, mice lacking myeloid lipin-1 enzymatic activity develop less atherosclerosis despite similar plasma lipid levels, supporting the conclusion that the enzymatic activity of macrophage-associated lipin-1 contributes to atherogenesis (158). Together, these studies support a model in which lipin-1 enzymatic activity can promote pro-inflammatory macrophage responses in settings of TLR activation and atherogenic lipid exposure. However, it is important to note that most studies to date have examined enzymatic activity under these inflammatory or lipid-loaded conditions, and its role in non-inflammatory or homeostatic macrophage states remains less well defined.

Although this area remains under-studied, emerging evidence supports an important role for the nonenzymatic activity of lipin-1, particularly in models of pro-resolving macrophage polarization. In IL-4-stimulated macrophages, loss of full lipin-1, but not selective loss of its enzymatic activity, impairs induction of wound-healing genes such as *Arg1, Socs2, Ccl17, Mannr, Il10*, and *Pparg*, indicating that nonenzymatic functions of lipin-1 contribute to optimal pro-resolving polarization in IL-4-driven models (35, 164, 165). Mechanistically, these findings are consistent with a model in which lipin-1 augments PPAR-dependent transcriptional programs while restraining transcription factors such as SREBP and NFAT, thereby favoring lipid catabolism, wound-healing gene expression, and the suppression of pro-inflammatory pathways rather than continued inflammatory activation (164, 165). *In vivo*, this nonenzymatic activity is associated with improved wound closure and increased CD206-expressing macrophages in wounds, and in atherosclerosis models it is linked to smaller plaques, reduced necrotic core formation, lower IL-23, less necroptosis, and more stable lesions, consistent with an atheroprotective role (164, 165). During pro-resolving responses, lipin-1 has been shown to act independently of its enzymatic activity in several experimental systems to reprogram macrophage metabolism in ways that support the resolution of inflammation. Mechanistically, loss of lipin-1 during pro-resolving responses leads to accumulation of citrate/isocitrate pathway intermediates, increased lipid synthesis, impaired efferocytosis, and delayed resolution of inflammation, whereas inhibition of mitochondrial citrate export restores efferocytosis and resolution in lipin-1-deficient macrophages and mice (35, 79). We have further shown that macrophage-associated lipin-1 promotes efferocytosis and inflammation resolution while restraining citrate-driven lipid synthesis, limiting neutral lipid and ceramide accumulation, and permitting phosphorylation-dependent inhibition of acetyl-CoA carboxylase, thereby shifting macrophages away from excess lipogenesis and toward β-oxidation and pro-resolving function (35, 79). By promoting efficient lipid utilization and restraining *de novo* fatty acid synthesis, macrophage-associated lipin-1 appears to limit ceramide-associated defects in pro-resolving macrophage function. Importantly, although these findings are consistent with a transcriptional coregulatory role for lipin-1, they do not yet establish that this is the sole nonenzymatic mechanism underlying these phenotypes. Other nonenzymatic mechanisms of lipin-1, including effects on RNA processing or membrane-contact-site organization, could also contribute. Collectively, these findings identify lipin-1 as an important organizer of macrophage lipid metabolism and support the need to define how lipin-1 determines when lipid synthesis is promoted versus restrained.

While these findings have led to a working model in which enzymatic activity of lipin-1 is associated with pro-inflammatory responses and nonenzymatic activity with pro-resolving functions, this distinction should be viewed with caution. Much of the current evidence derives from studies that examine enzymatic activity in inflammatory or lipid-loaded contexts, and nonenzymatic activity in IL-4-driven or wound-healing models. As a result, these functional assignments may reflect the experimental conditions under which lipin-1 has been studied rather than strict mechanistic separation of its activities. Notably, relatively little work has directly examined the role of lipin-1 enzymatic activity in non-inflammatory or pro-resolving settings, or conversely, the contribution of nonenzymatic functions during inflammatory activation. Addressing these gaps will be essential to determine whether lipin-1 functions are intrinsically partitioned or instead dynamically integrated across macrophage states. This further underscores the need for experimental approaches that directly disentangle lipin-1 enzymatic activity, transcriptional co-regulation, and other nonenzymatic mechanisms across diverse macrophage states.

Importantly, the observation that loss of full lipin-1 produces phenotypes not recapitulated by selective loss of enzymatic activity does not, by itself, establish that transcriptional coregulatory activity is the sole explanation. An important alternative possibility is that nonenzymatic functions of lipin-1 influence metabolic adaptation through additional mechanisms, including regulation of mRNA splicing fidelity or membrane-contact-site organization (166–168). In support of this possibility, during fasting adaptation in the liver, lipin-1 has been shown to interact with spliceosome proteins, maintain splicing fidelity during fasting in a PAP-independent manner, and regulate alternative splicing of metabolic transcripts, including genes involved in phospholipid metabolism (167). In parallel, recent work in non-macrophage systems suggests that lipin-1 can influence mitochondria-associated ER membrane (MAM) homeostasis, phospholipid organization, ER stress, Ca2+ transfer, and mitochondrial function, raising the possibility that some of the metabolic and pro-resolving phenotypes attributed to nonenzymatic lipin-1 may also reflect altered organelle communication rather than transcriptional control alone. In lipin-1-deficient macrophages undergoing pro-resolving responses, phosphatidylglycerol, the precursor of cardiolipin, is reduced (79, 169). Reduced phosphatidylglycerol abundance may be due, at least in part, to impaired ER–mitochondria lipid transfer associated with reduced MAM integrity (170, 171). Thus, while the current macrophage data are highly consistent with a transcriptional coregulatory model, they are better viewed as suggestive of a broader nonenzymatic regulatory role for lipin-1 that may encompass transcriptional coactivation, RNA processing, and membrane-organizational functions. Together, these findings support a model in which lipin-1 functions as a multi-modal regulator of macrophage metabolism, whose effects on inflammatory and pro-resolving responses emerge from the integration of enzymatic activity, transcriptional regulation, and organelle organization within broader cellular signaling networks.

## 5 Discussion

Macrophages must tightly regulate intracellular metabolism to maintain tissue homeostasis and support host responses. This review highlights the interconnected nature of macrophage lipid metabolism and shows how free fatty acid-derived pathways can have distinct, and sometimes opposing, effects on macrophage function. These pathways do more than provide substrates for energy production or membrane synthesis. They also generate metabolic intermediates and downstream lipid mediators, including prostaglandins and resolvins, that directly influence inflammatory activation, efferocytosis, and resolution. Overall, the studies reviewed here support a broader model in which macrophage function is shaped by how lipids are routed through competing metabolic fates. Fatty acids can be oxidized to support mitochondrial metabolism, incorporated into glycerolipids and lipid droplets for storage and buffering, used to generate inflammatory or pro-resolving lipid mediators, or diverted into sphingolipid pathways that promote lipotoxic stress. These pathways are not inherently inflammatory or pro-resolving. Instead, their functional consequences depend on macrophage activation state, tissue environment, lipid substrate, inflammatory stimulus, and disease stage. Thus, macrophage lipid metabolism should be viewed as a dynamic regulatory network that determines whether macrophages adapt to lipid burden, amplify inflammation, or support resolution.

This concept is especially relevant in cardiometabolic disease, where macrophages are chronically exposed to lipid-rich and inflammatory environments. In obesity, MASLD/MASH, and atherosclerosis, macrophage dysfunction is not driven simply by lipid excess, but by the failure to appropriately route fatty acids and lipid intermediates among oxidative, storage, signaling, and pro-resolving pathways. Although these diseases expose macrophages to distinct lipid environments, they share common challenges: buffering excess fatty acids, avoiding lipotoxic stress, preserving mitochondrial function, maintaining efferocytosis, and generating lipid mediators that promote resolution. ATMs must handle lipids released from stressed or dying adipocytes, hepatic macrophages encounter fatty acids, oxidized lipoproteins, and inflammatory signals derived from the gut and injured hepatocytes, and plaque macrophages process modified LDL, cholesterol, apoptotic cells, and inflammatory lipid mediators. In each setting, macrophage outcome depends not only on how much lipid is present, but on whether those lipids can be safely channeled into adaptive pathways.

These observations raise several unresolved biological questions. First, it remains unclear which lipid species are most responsible for macrophage dysfunction in specific cardiometabolic tissues. Ceramides, diacylglycerols, oxidized phospholipids, cholesterol derivatives, and saturated fatty acids have all been implicated in macrophage inflammatory activation, mitochondrial stress, impaired efferocytosis, and cell death, but their relative contributions likely differ by tissue, macrophage subset, and disease stage. Second, macrophage ontogeny may influence lipid handling. Tissue-resident macrophages and recruited monocyte-derived macrophages coexist in adipose tissue, liver, and atherosclerotic plaques, yet it remains unclear whether these populations differ in fatty acid uptake, intracellular trafficking, β-oxidation, lipid droplet storage, lipid mediator production, or susceptibility to lipotoxicity. Third, tissue-specific lipid niches likely shape macrophage metabolic behavior in ways that are not captured by standard *in vitro* polarization models. Future studies should therefore define how local lipid environments, macrophage origin, and inflammatory cues interact to determine lipid-routing decisions during disease.

These questions have direct therapeutic implications. Targeting lipid metabolic processes in cardiometabolic disease may provide opportunities to alter macrophage function, but these approaches will require greater precision than simply reducing lipid uptake or blocking lipid synthesis. In some settings, suppressing excessive fatty acid synthesis, DAG-dependent inflammatory signaling, ceramide production, or inflammatory eicosanoid generation may limit macrophage activation and tissue damage. In other settings, preserving lipid droplet buffering, mitochondrial β-oxidation, lipophagy, cholesterol efflux, PUFA mobilization, or SPM production may be necessary to support macrophage survival, efferocytosis, and resolution. Therefore, effective therapeutic strategies will likely need to restore balanced lipid channeling rather than inhibit a single lipid pathway in isolation. This is important because the same metabolic pathway may have different effects depending on timing and context. For example, lipid synthesis can support inflammatory mediator production, but basal lipid synthesis may also be required for efferocytosis. Likewise, β-oxidation may support pro-resolving functions, but it may also contribute to inflammatory responses under select conditions.

Within this framework, lipin-1 remains an important and instructive regulatory node. Lipin-1 is positioned at the intersection of phosphatidic acid (PA) metabolism, DAG production, glycerolipid synthesis, lipid storage, transcriptional regulation, mitochondrial metabolism, and inflammatory signaling. In inflammatory or lipid-loaded macrophages, lipin-1 enzymatic activity has been linked to DAG-dependent signaling pathways that amplify pro-inflammatory responses. In contrast, in pro-resolving macrophage models, lipin-1 appears to restrain excessive lipid synthesis, limit ceramide accumulation, preserve mitochondrial metabolism, and support efferocytosis. These findings suggest that lipin-1 does not function as a simple pro-inflammatory or pro-resolving switch. Rather, it acts as a context-dependent regulator of lipid routing whose effects depend on macrophage activation state, lipid environment, and disease setting.

Targeting lipin-1 therapeutically will therefore require careful attention to disease context, timing, and selectivity. In atherosclerosis, inhibiting lipin-1-dependent inflammatory DAG signaling in lipid-loaded plaque macrophages could reduce inflammatory activation, whereas preserving or enhancing lipin-1-dependent pro-resolving functions may support efferocytosis, necrotic core limitation, and plaque regression. In MASLD/MASH, modulation of lipin-1-linked lipid routing could influence whether hepatic macrophages buffer lipid stress or promote inflammation and fibrotic injury. In obesity, lipin-1 may help determine whether ATMs safely process excess lipid or adopt metabolically activated inflammatory states that contribute to insulin resistance. However, because lipin-1 also has important roles in systemic lipid homeostasis, skeletal muscle function, and energy balance, broad inhibition or activation may create unwanted effects. Future approaches should therefore prioritize macrophage-targeted delivery, disease-stage-specific intervention, and tools that distinguish lipin-1 enzymatic activity from nonenzymatic functions.

Overall, the next phase of work should move from describing lipid accumulation toward defining the metabolic decisions that control macrophage function in cardiometabolic disease. This will require integrated lipidomics, metabolic flux analysis, spatial approaches, genetic models, and selective pharmacologic tools that distinguish lipid uptake, storage, oxidation, mediator production, and lipotoxic signaling in defined macrophage populations. Within this broader effort, lipin-1 provides a useful model for understanding how lipid metabolic nodes coordinate macrophage inflammatory and pro-resolving functions. Defining when lipin-1 should be inhibited, preserved, or enhanced may help determine whether targeting macrophage lipid handling can improve outcomes in obesity, MASLD/MASH, atherosclerosis, and related cardiometabolic diseases.
